# Mitochondrial and Endoplasmic Reticulum Stress Trigger Triglyceride Accumulation in Models of Parkinson’s Disease Independent of Mutations in MAPT

**DOI:** 10.3390/metabo13010112

**Published:** 2023-01-09

**Authors:** Hugo J. R. Fernandes, Josh P. Kent, Michaela Bruntraeger, Andrew R. Bassett, Albert Koulman, Emmanouil Metzakopian, Stuart G. Snowden

**Affiliations:** 1 Department of Clinical Neurosciences, UK Dementia Research Institute, University of Cambridge, Cambridge Biomedical Campus, Cambridge CB2 0AH, UK; 2 Core Metabolomics and Lipidomics Laboratory, Institute of Metabolic Science, University of Cambridge, Level 4 Pathology, Cambridge Biomedical Campus, Cambridge CB2 0QQ, UK; 3 Wellcome Sanger Institute, Wellcome Genome Campus, Hinxton CB10 1SA, UK; 4 Department of Biological Sciences, Royal Holloway University of London, Egham, London TW20 0EX, UK

**Keywords:** metabolomics, Parkinson’s disease, triglyceride, LC-MS, mitochondrial stress, endoplasmic reticulum stress

## Abstract

The metabolic basis of Parkinson’s disease pathology is poorly understood. However, the involvement of mitochondrial and endoplasmic reticulum stress in dopamine neurons in disease aetiology is well established. We looked at the effect of rotenone- and tunicamycin-induced mitochondrial and ER stress on the metabolism of wild type and microtubule-associated protein tau mutant dopamine neurons. Dopamine neurons derived from human isolated iPSCs were subjected to mitochondrial and ER stress using RT and TM, respectively. Comprehensive metabolite profiles were generated using a split phase extraction analysed by reversed phase lipidomics whilst the aqueous phase was measured using HILIC metabolomics. Mitochondrial and ER stress were both shown to cause significant dysregulation of metabolism with RT-induced stress producing a larger shift in the metabolic profile of both wild type and MAPT neurons. Detailed analysis showed that accumulation of triglycerides was a significant driver of metabolic dysregulation in response to both stresses in both genotypes. Whilst the consequence is similar, the mechanisms by which triglyceride accumulation occurs in dopamine neurons in response to mitochondrial and ER stress are very different. Thus, improving our understanding of how these mechanisms drive the observed triglyceride accumulation can potentially open up new therapeutic avenues.

## 1. Introduction

Currently it is estimated that there are almost 150,000 Parkinson’s disease (PD) sufferers in the UK [1]. With age representing the biggest risk factor for disease onset, with only 4% of sufferers under the age of 50, the prevalence of PD is expected to increase significantly in the future with an estimated 265,000 sufferers by 2065 [1].

The exact causes of PD remain unknown; however, it is thought that a complex set of interactions between genetic and environmental factors are responsible for the development of the disease [2,3,4]. The pathology of PD is characterised by the progressive degeneration of dopaminergic neurons in the substantia nigra causing a deficiency of dopamine in the striatum. The cause of this loss of dopamine neurons is poorly understood but has been shown to be associated with intra-cellular accumulation of misfolded proteins [2,5]. In addition to this, studies in humans and PD models have also implicated mitochondrial and ER dysfunction in its pathology [6,7].

The brain is the most energy demanding part of the body, accounting for about 20% of total demand, with the mitochondrial oxidative phosphorylation and electron transport chain (ETC) playing key roles of ATP synthesis. PD is strongly associated with mitochondrial dysfunction, in particular with the loss of function of complex 1 of the ETC [8,9]. The majority of substrates for oxidative phosphorylation enters the ETC via complex 1, also known as NADH-quinone oxidoreductase, and its inhibition leads to reduced production of ATP and loss of bioenergetics function in the cell [8].

Accumulation of misfolded proteins is a central paradigm in PD pathology, and because of its key role in protein homeostasis and folding understanding the role of the ER is crucial [10]. In PD, α-synuclein is the main constituent of protein aggregates and has been shown to be toxic and lead to ER stress [10] triggering the unfolded protein response (UPR) leading to neuronal loss. Thayanidhi et al. showed that overexpression of ER to Golgi transport suppressed toxicity of α-synuclein by reducing misfolding and subsequent aggregation [11].

The microtubule-associated protein tau (MAPT) gene encodes the protein Tau that aggregate to form neurofibrillary tangles which are core pathological feature of Alzheimer’s disease but have also been identified in PD [12,13]. Several previous studies have shown that mutations in this gene, especially the H1 haplotype have been shown to associate with the age of onset and rate of decline in PD patients [14,15,16].

In this study, we explored how the effect of PD associated mitochondrial and ER stress on the metabolism of human-derived dopamine neurons and how mutations of the MAPT gene modulate these metabolic responses, to determine if these mutations exacerbated the effects of cellular stress on metabolism. To investigate this, we used Rotenone (RT) to inhibit complex 1 of the mitochondrial ETC, and Tunicamycin (TM) to inhibit protein glycolysis and induce ER stress. Mitochondrial dysfunction and mitochondrial oxidative stress are hallmarks of PD and a common feature described in animal and cell models of PD. To induce this perturbation in our model, we treated our dopaminergic neurons with rotenone, a toxic pesticide known to cause PD-like symptoms in humans and animal models [17,18]. Rotenone induces in vivo loss of substantia nigra dopaminergic neurons (a hallmark of PD neuropathology) and causes aggregation of alpha-synuclein, a major component of Lewy bodies protein aggregates found in PD patient brains. The endoplasmic reticulum (ER) is a central organelle for protein folding, lipid synthesis, and calcium storage. Disturbances in ER homeostasis can be caused by PD associated perturbations including accumulation of misfolded proteins, oxidative stress, and calcium imbalance, resulting in ER stress. ER stress has been described in a variety of PD models, including post-mortem brain tissue from sporadic disease [19], toxin models of PD [20,21], a yeast alpha-synucleopathy model [22], A53T mouse and rat PD models [23] and in iPSC-derived neuronal models from PD patients [7]. Taken together, these and other reports suggest a central role for ER stress in PD but the precise mechanisms leading to pathogenesis are still unclear.

## 2. Materials and Methods

### 2.1. Cell Culture and Dopaminergic Differentiation

Human iPSCs were cultured in TeSR-E8 medium on Vitronectin coated plates. Cells were passaged with 0.5 mM EDTA when reaching 70% confluency at a ratio of 1:6. Differentiation into dopaminergic neurons was performed according modify version of existing experimental protocols [24,25]. In brief, iPSCs were first dissociated into single cells, plated at 150,000 cells/cm^2^ on Geltrex coated plates and grown for 11 days in Knockout Serum Replacement media (KSR) containing KO DMEM media, KSR (15%), Nonessential Amino Acids (1:100), 2-Mercaptoethanol (10 μM) and 2 mM L-glutamine. KSR medium was gradually changed to NNB medium containing Neurobasal medium, N2 (0.5X) and B27 (0.5X) and 2 mM L-glutamine from day 6. Media was changed to NB medium on day 12 containing Neurobasal medium, B27 (1X) and 2 mM L-glutamine. Medias were supplemented with LDN-193189 (100 nM) from days 0–10; SB431542 (10 μM) from days 0–4; SAG (100 nM) from days 1–6; Purmorphamine (2 μM) from days 1–6; FGF8a (100 ng/mL) from days 1–6; and CHIR99021 (3 μM) from days 3–12. From Day 12 onwards, the following supplements were added: BDNF (20 ng/mL), GDNF (20 ng/mL), Ascorbic Acid (200 μM), TGFβ3 (1 ng/mL), dibutyryl cAMP (500 μM), and DAPT (10 μM). At day 21 cells were dissociated with StemPro Accutase and replated at 300,000 cells/cm^2^ in dishes pre-coated with Geltrex and fed every second day for 2 weeks before analysis.

### 2.2. Drug Treatments

After differentiation into dopaminergic neurons, cells were treated for 24 h with either 5 μM of Tunicamycin or 1μM of Rotenone [26].

### 2.3. Generation of Isogenic Heterozygous and Homozygous MAPT N279K hiPSCs

Human pluripotent stem cell lines used in this study were KOLF2-C1 (WTSIi018-B-1) generated under London Fulham REC Reference 14/LO/0345). Optimal gRNA design was performed using the WGE tool (https://wge.stemcell.sanger.ac.uk/, accessed on 9 December 2021) to minimise off target effects. Full length chemically modified sgRNA was purchased (Synthego) with the MAPT-targeting gRNA sequence: TTATTAATTATCTGCACCTT TGG. CRISPR/Cas9 genome editing was performed as previously described [27]. Briefly, eSpCas9_1.1/sgRNA ribonucleoprotein complexes and ssODN (tggcgtgtcactcatccttttttctggctaccaaagGTGCAGATAATTAAgAAGAAGCTGGATCTTAGCAACGTCCAGTCCAAGTGTGGCTCAAAGGATA, IDT Ultramer) were transfected into iPSCs by electroporation using nucleofection according to the manufacturer’s instructions (Lonza 4D nucleofector, CA137, P3 buffer). Cells were allowed to grow to 75% confluence, dissociated using Accutase and plated onto a Synthemax II-SC-coated 10 cm^2^ dish (Corning) at a low density. Individual colonies were picked manually into 96 well plates coated in matrigel (Thermo Scientific, Loughborough, UK) for expansion and MAPT-N279K clones were identified by high throughput sequencing. Sequencing was performed from PCR products using primers (F-ACACTCTTTCCCTACACGACGCTCTTCCGATCTgcatgtcactcatcgaaagtggag, R-TCGGCATTCCTGCTGAACCGCTCTTCCGATCTctaataattcaagccacagcacggcg), indexed and sequenced on an Illumina MiSeq instrument, and final clones validated by Sanger sequencing of PCR products (F-ctcaggctggtgaacgctccc, R-tcctagaatatgaggaaggggcttctgg.

### 2.4. Chemicals and Reagents

All solvents and reagents used for LC-MS analysis water, methanol, acetonitrile, methyl-tertiary butyl ether (MTBE) and ammonium formate were all LC-MS grade and purchased from either Sigma-Aldrich (Gillingham, UK) or Fisher Scientific (Loughborough, UK). Two internal standards were added to all samples L-valine ^13^C_5_^15^N (95%) and tripentadecanoin purchased from Sigma-Aldrich (Gillingham, UK).

### 2.5. Sample Preparation

Sample extraction was performed using a modified version of an in-vial dual extraction described previously [28]. Briefly, 5 µL of HILIC internal standard (2.5 mM L-valine ^13^C_5_^15^N in 80:20 MeOH:H_2_O), 140 µL of MTBE containing 15 µM of tripentadecanoin and 40 µL of methanol were added to all cell pellets and left to stand for 10 min to disrupt the cell membranes. After this samples were transferred to a HPLC vial with a 250 µL glass insert and 30 µL of water containing 0.15 mM ammonium formate was added before the was sample spun at 5000× *g* for 5 min to achieve phase separation. Extraction blanks were obtained by extracting 15 µL of HPLC grade water using the same protocol, 5 µL of all analytical samples was pooled to create quality controls.

### 2.6. LC-MS Analysis of Aqueous Phase (HILIC)

LC-MS analysis was performed on an Agilent infinity HPLC system coupled to and Agilent 6550 ion funnel QToF (Agilent, Santa Clara, CA, USA). Separation of the aqueous phase metabolites was performed on Agilent Poroshell HILIC-z column (2.1 × 150 mm, 2.7 μM) using 10 mM ammonium formate in water as mobile phase A and 2.5 mM ammonium formate in acetonitrile as mobile phase B. The column temperature was set to 30 °C with a flow rate of 0.25 mL/min, the gradient was held isocratic for the first minute at 5% mobile phase A prior to a linear increase to 10% at 6 min and 25% at 15 min after which there was a 3 min column washing step at 80% mobile phase A before initial conditions were restored to allow 7 min for column re-equilibration. Data was collected between 50–1000 *m*/*z*, with a gas temperature of 200 °C, a drying gas flow of 15 L/min, a nebulizer pressure of 40 psi a sheath gas flow of 12 L/min and temperature of 300 °C.

### 2.7. LC-MS Analysis of Non-Aqueous Phase (Reversed Phase)

Separation of the aqueous phase metabolites was performed on Agilent Poroshell C18 column (2.1 × 150 mm, 2.7 μM) using 10 mM ammonium formate in water as mobile phase A and 10 mM ammonium formate in methanol:MTBE (2:1 v:v) as mobile phase B. The column temperature was set to 55 °C with a flow rate of 0.425 mL/min, the gradient started out at 20% mobile phase A before a linear decrease to 7% at 13 min, 6% at 20 min and 4% at 24 min prior to a 6 min washing step of 100% mobile phase B prior to the restoration of initial conditions to allow 5 min of re-equilibration. Data was collected between 50–1200 *m*/*z*, with a gas temperature of 200 °C, a drying gas flow of 15 L/min, a nebulizer pressure of 35 psi a sheath gas flow of 10 L/min and temperature of 120 °C.

### 2.8. Data Processing and Statistical Analysis

All .d files generated by the MS were converted into .mzXML files using Proteowizard [29]. Converted data files were processed using the CAMERA package performed in the open-source software package R (v3.6.0), with peak picking performed using a “centwave” method which allows for the deconvolution of closely eluting or slightly overlapping peaks [30]. After this had been done, metabolite features were defined as any peak with an average intensity at least 5 times higher in analytical samples relative to the abundance seen in the extraction blanks, with the peak having to be present in all of the samples of at least one sample group.

The combined datasets (both HILIC and RP data) were analysed using a range of multivariate algorithms including principle component analysis (PCA) and partial least squares discriminant analysis (PLS-DA) performed in SIMCA (v13.0.4) and with all data logarithmically transformed (base10) and scaled to unit variance (UV). Model performance was assessed based on the cumulative correlation coefficients (R^2^X[cum]) and predictive performance based on 7-fold cross validation (Q^2^[cum]), with the significance of the model assessed based on the ANOVA of the cross-validated residuals (CV-ANOVA). Univariate analysis to identify individual metabolite variables that differed between groups was performed using generalised linear models (GLM) performed in R (v3.6.0). All metabolites were annotated by matching metabolite fragmentation spectra to spectra held in in-house and publicly available spectral libraries including HMDB (hmdb.ca) and METLIN (metlin.scripps.edu).

## 3. Results

**Figure 1:**
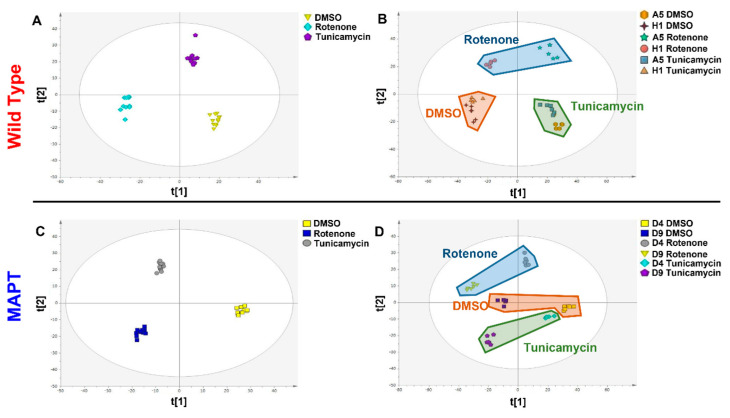
Multivariate partial least squares−discriminant analysis (PLS−DA) of Rotenone and Tunicamycin treatment in wild type and MAPT mutant dopamine neurons. (**A**) PLS-DA scores plot of combined treatment groups in wild type neurons (t[1] = 16.4% and t[2] = 7.7%) (**B**) PLS-DA scores plot of treatment response in individual wild type cell lines (t[1] = 38.3% and t[2] = 15.4%) (**C**) PLS-DA scores plot of combined treatment groups (t[1] = 18.2% and t[2] = 8.9%) (**D**) PLS-DA scores plot of treatment response in individual MAPT cell lines (t[1] = 38.6% and t[2] = 7.9%). Abbreviations: MAPT; microtubule associated protein tau.

In this study, a total of 6359 metabolite features were measured, with 3589 measured from the aqueous fraction and 2770 from the organic phase. Partial least square–discriminant analysis was applied to genotypes independently with samples grouped based on cell line and treated and calculated using all metabolite features. This showed that both RT and TM treatment are modifying the metabolism of wild type dopamine neurons derived from human iPSC (Figure 1A, R^2^X = 0.241 R^2^Y = 0.923 Q^2^ = 0.828 CV-ANOVA = 9.91 × 10^−19^). When looking at individual cell lines (Figure 1B, R^2^X = 0.537 R^2^Y = 0.603 Q^2^ = 0.488 CV-ANOVA = 4.34 × 10^−5^) it can be seen that whilst there are differences in the metabolite composition of the lines (seen on the first component) the treatment is having similar metabolic effects in both lines with RT having the largest effect. When looking at the effect of these treatments on MAPT mutant dopamine neurons a similar response can be seen to that observed in wild type cells with both treatments causing significant modifications in metabolism (Figure 1C, R^2^X = 0.271 R^2^Y = 0.625 Q^2^ = 0.387 CV-ANOVA = 0.0011). When looking at individual cell lines it can again be seen that whilst there are differences in metabolite composition between the lines but that they exhibit similar responses to treatment with RT again having the largest effect (Figure 1D, R^2^X = 0.465 R^2^Y = 0.975 Q^2^ = 0.460 CV-ANOVA = 6.03 × 10^−5^).

Univariate analysis using generalised linear models comparing all wild type DMSO and RT treated cells identified that 838 metabolite features were significantly (*p* < 0.05) associated with mitochondrial with 142 passing FDR correction based on Bejamini-Hochberg based on all 6359 metabolite features with 36 successfully annotated (Table 1).

When looking at the metabolite features associated with ER stress 947 were shown to be significantly (*p* < 0.05) associated with TM treatment (*p* < 0.05) with 28 passing FDR correction with 18 being annotated (Table 2).

When looking at overlap of metabolic response to mitochondrial and ER stress in wild type neurons, the shift in 287 features was conserved whilst the change in 550 metabolite features were unique to RT treatment and 660 were unique to TM. In MAPT mutant neurons, 285 were common between the treatments with 592 specific to mitochondrial stress and 337 unique to ER stress. Looking at overlap of metabolic response to mitochondrial stress, shifts in the abundance of 396 metabolite features were common to both genotypes whilst 439 were unique to the wild type and 481 were specific to MAPT mutants. In response to ER stress, the shifts in 185 metabolite features were common between genotypes with 762 specific to wild type and 437 unique to MAPT cells.

**Figure 2:**
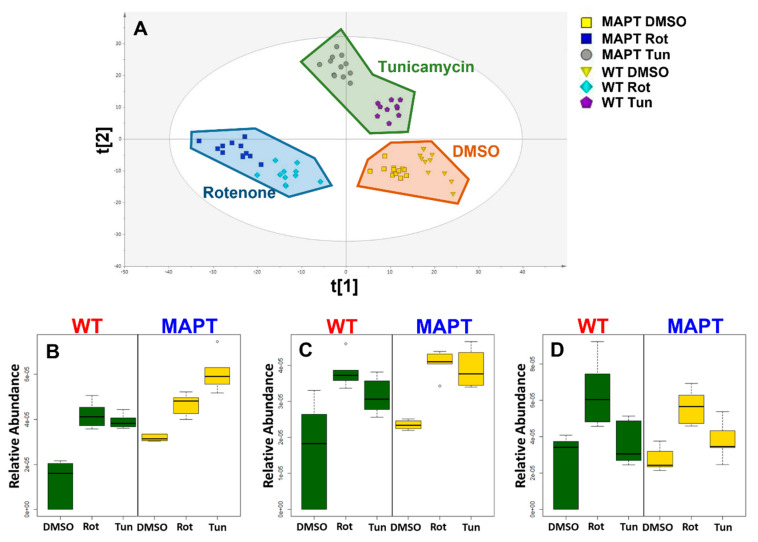
Multivariate and univariate analysis of triglycerides in response to Rotenone and Tunicamycin in both wild type and MAPT mutant dopamine neurons. (**A**) PLS−DA scores plot calculate from triglyceride data for all groups (t[1] = 31.1% and t[2] = 11.1%) (**B**) boxplot of TG(54:2), (**C**) boxplot of TG(58:3), (**D**) boxplot of TG(58:5). Abbreviations: MAPT; microtubule associated protein tau, Rot; rotenone, TG; triglyceride, Tun; tunicamycin, WT; wild type.

We found that both mitochondrial and ER stress caused significant dysregulation of triglyceride (TG) metabolism, and when we look at the inter-relationship between treatment groups based solely on annotated triglycerides profound differences were still observed, with the PLS-DA model calculated using only the TGs from Table 3 with samples grouped by genotype and treatment (Figure 2A R^2^X = 0.422 R^2^Y = 0.511 Q^2^ = 0.428 CV-ANOVA = 3.22 × 10^−6^).

In wild type cells, 20 and 14 TGs were significantly associated with RT and TM, respectively, with 13 common to both treatments with all species increased in abundance by stress (Table 3). In MAPT cells, 14 TGs were associated with both treatments with 21 and 15 specifically accumulating after RT and TM (Table 3). Looking at the overlap between genotypes 20 RT associated TGs were common to both genotypes whilst 11 of TM associated species were common between wild type and MAPT cells.

## 4. Discussion

Mitochondrial and ER stress and specifically RT and TM treatment have previously been shown to cause significant metabolic dysregulation and intracellular accumulation of triglyceride species as is observed in this study [31,32,33,34]. However, analysis of the data in this study it appears that whilst both treatments are producing similar metabolic phenotypes the mechanisms by which these compounds achieve this may be very different.

Intracellular accumulation of triglycerides in response to mitochondrial dysfunction is well documented [35,36]; however, the mechanisms driving this is not fully understood. Vankoningsloo et al. suggested that a reduction in fatty acid β-oxidation and increased glucose uptake drives TG accumulation [34]. They demonstrated that inhibition of the electron transport chain with antimycin A increased TG levels but did not increase the uptake TG precursors but found that the rate of β-oxidation was decreased in pre-adipocytes [34]. This suggests that the accumulation of TGs in response to ETC inhibition is being driven by reduced breakdown rather than increased production. However, glucose is the main energy source for both neuronal and glial cells [37,38], in contrast to other tissues with high energy demand, such as the heart and kidney, which obtain between 60 and 80% of their energy from β-oxidation of long chain fatty acids (LCFAs). β-oxidation only plays a minor role in brain energy metabolism [39]. Several reasons have been posited for this including difficulty in transporting FAs across the blood–brain barrier, low enzymatic capacity to degrade FAs and side effects of none esterified LCFAs. It is possible that this reliance on glucose of neuronal and glial cells is caused by the availability of the substrate and that when exposed to more readily available FAs, such as in culture conditions, the cells revert to B-oxidation, but there is no clear proof of this.

**Figure 3:**
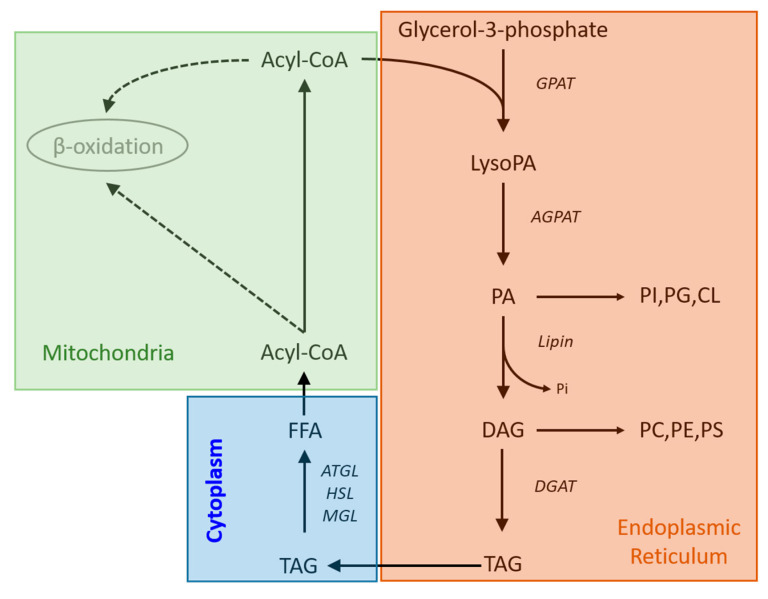
Metabolic pathways showing the routes of triglyceride synthesis and breakdown. Abbreviations: AGPAT; acylglycerol-phosphate-acyltransferase, ATGL; adipose triglyceride lipase, CL; cardiolipin, DGAT; diglyceride acyltransferase, GPAT; glycerol-phosphate-acyltransferase, HSL; hormone sensitive lipase, LysoPA; lysophosphatidyladipic acid, MGL; monoglyceride lipase, PA, phosphatidic acid, PC; phosphatidylcholine, PE; phosphatidylethanolamine, PG; phosphatidylglycerol, PI; phosphatidylinositol, PS; phosphatidylserine.

Therefore, it would suggest that the TG accumulation in human dopamine neurons after RT treatment observed in this study is not the result of a reduction in the rate of β-oxidation as the basal rate of this process is so low. However, inhibition of mitochondrial complex 1 by RT, can activate de novo synthesis of fatty acids and TG production [32]. It potentially does this by increasing the expression and concentration of glycerol phosphate acyltransferase (GPAT) which converts acyl-CoA to acyl-glycerol-phosphate and has been shown to be the rate limiting step in TG synthesis [40]. Taken together the results of this study and the literature suggest that inhibition of the ETC could reduce the use of acyl-CoA by β-oxidation leading to decreased TG breakdown and providing a larger pool of precursor for unregulated GPAT to metabolise, leading to a profound TG accumulation (Figure 3). Our data showed that RT is having a similar effect on the metabolism of both WT and MAPT neurons with 20 and 21 TGs shown to accumulate significantly in both genotypes, respectively, with no difference in the degree of accumulation. This suggests that whilst PD associated mitochondrial stress is causing TG accumulation this effect is independent of mutations in the MAPT gene.

Fuchs et al. induced ER stress in cultured hepatic cells (Hepa1.6) using TM and found that TGs accumulated intracellularly, and that overexpression of adipose triglyceride lipase (which would increase TG breakdown) increased ER stress [31]. The relationship between ER stress and lipid metabolism has been shown to be bidirectional, and whilst activation of ER stress pathways can result in lipogenesis and altered lipid homeostasis, lipids and aberrant lipid metabolism can also cause ER stress via lipotoxicity [41,42,43,44]. We have previously shown that the specific A53T mutation in the alpha-synuclein gene can lead to a reduced heterogeneity of lipid metabolism in dopamine neurons [45], further contributing to their susceptibility to lipotoxicity.

In this study, we showed increased levels of diglycerides, and phospholipids in both WT and MAPT neurons (Table 2), and these specific lipids have all been shown to cause lipotoxicity, with these lipids shown to correlate with the degree of cellular dysfunction [46,47,48]. Whilst from this data we cannot know exactly what fraction of the phospholipids are free in the cell and how much is bound in membranes it is likely that the relative fraction is the same in both DMSO- and TM-treated cells meaning that the levels of free lipids is higher in TM-treated cells and thus lipotoxicity is increased. It has been shown that storing these lipotoxic intermediates in the form of TGs can help to limit the build-up of these intermediates, especially DGs and ceramides reducing stress on the ER [46,47,48]. These findings suggest that the increase in TGs that we observed is an adaptive response to the lipotoxicity driving ER stress. Interestingly ER stress has also been shown to increase lipolysis by activating cAMP/PKA and ERK1/2 [49], potentially producing lipotoxic free fatty acids and mitigating the adaptive response of storing lipids as neutral TGs [50]. This lipotoxicity could lead to disruption of ER function causing disruption of protein folding, which activates the unfolded protein response (UPR). Initially UPR aims to restore ER homeostasis, and if this cannot be achieved it drives cellular apoptosis [51]. With this potential increase in TG turnover, with both production and breakdown of TGs, future studies looking at the rate of turnover of these molecules is required to fully understand their role on PD pathology. In this study, we saw on average there was a 106% greater accumulation of TGs in response to TM treatment in MAPT cells relative to WT, suggesting that these MAPT dopamine neurons are more susceptible to ER stress thus causing increased ER dysfunction. This greater susceptibility is potentially the result of the mitochondria associated ER membranes with pathogenic expression of MAPT resulting in mitochondrial disruption, transport and clearance [52,53,54] with recent reports suggests that mutated tau impairs mitophagy [54,55,56] subsequently effecting ER function. The differences resulting from the MAPT mutation are not seen in response to rotenone treatment as the mitochondria will be stressed equally in both genotypes. Tunicamycin treatment will stress the ER in both genotypes; however, mutant MAPT cells will also experience mitochondrial stress as a result of mutation potentially explaining the greater TG accumulation. In future, it is important to determine if similar accumulation of TGs in tissue of deceased PD patients can be confirmed. This provides further evidence that reducing lipotoxic stress could help to preserve ER function in PD patients especially those that have mutations of the MAPT gene.

## 5. Conclusions

In this study, we demonstrated that simulation of the mitochondrial and ER stress observed in PD caused significant accumulation of TGs in human-derived dopamine neurons with mutations in the MAPT gene appearing to exacerbate the effect of ER stress. Whilst previous studies have postulated a range of mechanisms by which RT and TM can cause this accumulation it is unclear whether this build up is the result of increased production or reduced breakdown to TGs. We would suggest that future studies use fluxomics approaches to directly determine the relative rates synthesis and breakdown of these molecules to determine how mitochondrial and ER stress are driving dysregulation of TG metabolism and to identify potential therapeutic targets.

## Figures and Tables

**Table 1:** Annotated metabolites associated with rotenone treatment in both wild type and MAPT mutant dopamine neurons.

	Wild Type			MAPT		
	*p*-Value	q-Value	FC^+^	*p*-Value	q-Value	FC^+^
Hydroxybutyrylcarnitine	5.84 × 10^−9^	1.86 × 10^−5^	10.37	6.59 × 10^−5^	0.0029	7.00
TG(54:7)	5.86 × 10^−7^	0.0009	2.45	2.57 × 10^−8^	8.6 × 10^−6^	2.36
TG(56:6)	3.42 × 10^−6^	0.0025	6.04	4.36 × 10^−5^	0.0021	7.53
TG(58:7)	3.48 × 10^−6^	0.0025	2.17	0.0004	0.012	1.99
TG(54:2)	4.37 × 10^−6^	0.0027	1.97	6.84 × 10^−5^	0.0029	2.65
PE(36:0)	6.60 × 10^−6^	0.0034	1.07	0.381	0.799	1.23
PC(34:6)	6.86 × 10^−6^	0.0034	0.91	0.689	0.845	1.52
PE(38:2)	8.36 × 10^−6^	0.0035	2.35	7.12 × 10^−13^	1.94 × 10^−9^	2.48
TG(61:7)	9.61 × 10^−6^	0.0036	3.82	1.21 × 10^−7^	2.41 × 10^−5^	5.44
TG(52:2)	9.62 × 10^−6^	0.0036	3.05	0.0002	0.0078	4.20
LysoPC(18:2)	1.85 × 10^−5^	0.0049	1.28	4.40 × 10^−5^	0.0021	1.44
TG(58:5)	1.85 × 10^−5^	0.0049	1.82	7.32 × 10^−6^	0.0005	2.11
SM(38:6)	1.92 × 10^−5^	0.0049	2.12	0.011	0.151	2.70
DG(38:5)	2.26 × 10^−5^	0.0053	3.34	6.56 × 10^−7^	9.07 × 10^−5^	3.28
TG(60:9)	3.05 × 10^−5^	0.0065	3.01	5.59 × 10^−7^	8.01 × 10^−5^	3.97
TG(61:6)	3.77 × 10^−5^	0.0074	4.40	1.48 × 10^−9^	1.08 × 10^−6^	4.04
TG(59:5)	4.93 × 10^−5^	0.0085	4.09	9.53 × 10^−7^	0.0001	3.36
TG(58:3)	5.08 × 10^−5^	0.0085	1.57	0.164	0.605	3.99
Pantothenamide	6.21 × 10^−5^	0.0096	0.02	4.82 × 10^−7^	7.3 × 10^−5^	0.02
TG(57:4)	6.45 × 10^−5^	0.0098	2.63	9.68 × 10^−7^	0.000118	3.14
TG(51:2)	6.81 × 10^−5^	0.0099	2.14	0.0035	0.064	3.78
PC(40:8)	8.5 × 10^−5^	0.011	2.02	3.73 × 10^−5^	0.0018	2.19
TG(62:10)	8.63 × 10^−5^	0.011	3.19	0.0002	0.0073	3.32
Tocotrienol	0.0002	0.019	1.13	0.464	0.564	2.18
TG(61:5)	0.0002	0.020	2.86	2.85 × 10^−8^	9.05 × 10^−6^	2.88
TG(52:7)	0.0003	0.022	2.16	0.0015	0.033	2.30
Triglylcarnitine	0.0003	0.023	0.00	2.21 × 10^−5^	0.0012	0.01
LysoPC(18:3)	0.0003	0.024	1.23	0.0012	0.028	1.35
Hexadecenoylcarnitine	0.0004	0.024	0.90	0.410	0.812	1.57
Cer(40:2)	0.0004	0.025	1.26	0.148	0.579	1.82
Acetylputrescine	0.0006	0.035	0.42	2.25 × 10^−5^	0.0012	0.42
TG(54:3)	0.0007	0.037	4.10	2.10 × 10^−8^	7.41 × 10^−6^	3.93
Propionylcarnitine	0.0008	0.039	0.05	3.94 × 10^−6^	0.0003	0.04
Tetrahydropterin	0.0008	0.039	0.67	0.034	0.297	1.85
Leucine/Isoleucine	0.0010	0.047	0.20	0.0024	0.049	0.02
DG(38:4)	0.0011	0.049	0.78	0.910	0.954	1.52

All *p*-values calculated using generalized liner models, corrected q-values calculated using benjamini-hochberg, ^+^ fold change calculated relative to DMSO treated samples. Abbreviations: Cer; ceramide, DG; diglyceride, FC; fold change, LysoPC; lysophosphatidylcholine, PC; phosphatidylcholine, PE; phosphatidylethanolamine, SM; sphingomyelin, TG; triglyceride.

**Table 2:** Annotated metabolites associated with tunicamycin treatment in both wild type and MAPT mutant dopamine neurons.

	Wild Type			MAPT		
	*p*-Value	q-Value	FC^+^	*p*-Value	q-Value	FC^+^
PC-O(38:3)	1.42 × 10^−7^	0.0003	1.41	0.016	0.306	1.32
TG(58:3)	1.21 × 10^−6^	0.0019	2.07	0.0030	0.117	4.11
TG(54:2)	2.91 × 10^−5^	0.0021	2.24	0.0001	0.018	2.46
CE(20:4)	4.50 × 10^−5^	0.0030	3.02	0.0010	0.056	3.45
PIP(34:1)	6.86 × 10^−5^	0.0050	1.12	0.207	0.748	1.45
PI(40:6)	0.0001	0.0050	1.08	0.125	0.666	1.25
PE(36:0)	0.0002	0.0074	1.04	0.660	0.726	1.13
PI(34:2)	0.0002	0.0082	0.45	0.0006	0.039	0.58
DG(46:7)	0.0002	0.0097	1.17	0.044	0.619	1.22
TG(52:2)	0.0003	0.013	2.15	0.033	0.438	2.84
PE(40:4)	0.0003	0.013	1.04	0.109	0.646	1.10
PS(42:6)	0.0004	0.013	1.09	0.085	0.605	1.23
TG(54:3)	0.0006	0.019	2.74	0.0056	0.164	2.99
IMP	0.0010	0.019	0.84	0.818	0.916	0.09
TG(58:5)	0.0010	0.019	1.69	0.0001	0.020	1.68
TG(58:8)	0.0011	0.021	1.39	0.303	0.819	2.49
TG(56:6)	0.0022	0.022	2.82	0.0015	0.076	2.55
TG(58:7)	0.0042	0.022	1.59	0.021	0.351	1.51

All *p*-values calculated using generalized liner models, corrected q-values calculated using benjamini-hochberg, ^+^ fold change calculated relative to DMSO treated samples. Abbreviations: CE; cholesterylester, DG; diglyceride, FC; fold change, IMP; inosine monophosphate, LysoPC; lysophosphatidylcholine, PC; phosphatidylcholine, PE; phosphatidylethanolamine, PI; phosphatidylinositol, PS; phosphatidylserine, TG; triglyceride.

**Table 3:** Annotated triglycerides that accumulate in response to Rotenone and Tunicamycin treatment in both wild type and MAPT mutant dopamine neurons.

	DMSO vs. Rotenone				DMSO vs. Tunicamycin			
	Wild Type		MAPT		Wild Type		MAPT	
	*p*-Value	FC+	*p*-Value	FC+	*p*-Value	FC+	*p*-Value	FC+
TG(61:6)	1.48 × 10^−9^	4.44	3.77 × 10^−5^	4.04	0.0085	1.87	0.035	4.04
TG(54:3)	2.10 × 10^−8^	4.09	0.0007	3.93	0.0056	2.74	0.0006	3.93
TG(54:7)	2.57 × 10^−8^	2.45	5.86 × 10^−7^	2.36	0.497	1.14	0.012	2.36
TG(61:5)	2.85 × 10^−8^	2.86	0.0002	2.88	0.0009	1.88	0.0095	2.88
TG(61:7)	1.21 × 10^−7^	3.82	9.61 × 10^−6^	5.44	0.207	1.33	0.032	5.44
TG(59:3)	3.74 × 10^−7^	1.96	0.0008	2.74	0.0022	1.55	0.0067	2.74
TG(59:4)	5.56 × 10^−7^	2.68	0.0003	3.09	0.0002	1.49	0.019	3.09
TG(60:9)	5.59 × 10^−7^	3.01	3.05 × 10^−5^	3.97	0.159	1.20	0.018	3.97
TG(59:5)	9.53 × 10^−7^	4.09	4.93 × 10^−5^	3.36	0.014	1.63	0.866	3.36
TG(57:4)	9.68 × 10^−7^	2.63	6.45 × 10^−5^	3.14	0.029	1.34	0.922	3.14
TG(57:3)	1.56 × 10^−6^	2.17	0.0004	2.40	0.0059	1.41	0.294	2.4
TG(58:5)	7.32 × 10^−6^	1.82	1.85 × 10^−5^	2.11	0.0001	1.69	0.001	2.11
TG(60:6)	7.40 × 10^−6^	2.49	0.0006	2.69	0.094	1.44	0.038	2.69
TG(56:6)	4.36 × 10^−5^	6.04	3.42 × 10^−6^	7.53	0.0015	2.82	0.0023	7.53
TG(54:2)	6.84 × 10^−5^	1.97	4.37 × 10^−6^	2.65	0.0001	2.24	2.91 × 10^−5^	2.65
TG(62:10)	0.0002	3.19	8.63 × 10^−5^	3.32	0.519	1.35	0.339	3.32
TG(52:2)	0.0002	3.05	9.62 × 10^−6^	4.20	0.033	2.15	0.0003	4.2
TG(58:7)	0.0004	2.17	3.48 × 10^−6^	1.99	0.021	1.59	0.0042	1.99
TG(52:7)	0.0015	2.16	0.0003	2.30	0.105	0.80	0.196	2.3
TG(51:2)	0.0035	2.14	6.81 × 10^−5^	3.78	0.485	1.15	0.089	3.78
TG(58:3)	0.164	1.57	5.08 × 10^−5^	3.99	0.0030	2.07	1.21 × 10^−6^	3.99

All *p*-values calculated using generalized liner models, ^+^ fold change calculated relative to DMSO treated samples. Abbreviations: DMSO; dimethylsulfoxide, FC; fold change, TG; triglyceride.

## Data Availability

All processed data will be available on request from the corresponding author. Data is not publicly available due to privacy or ethical restrictions.
